# The Karolinska experience of autologous stem-cell transplantation for lymphoma: a population-based study of all 433 patients 1994–2016

**DOI:** 10.1186/s40164-019-0131-3

**Published:** 2019-03-18

**Authors:** Mattias Carlsten, Martin Jädersten, Anna Hellström, Karin Littmann, Christopher M. Melén, Henna Riikka Junlén, Kristina Sonnevi, Per Ljungman, Bo Björkstrand, Björn Engelbrekt Wahlin

**Affiliations:** 1 Center for Hematology and Regenerative Medicine, Dept. of Medicine, Huddinge, Karolinska Institutet, Stockholm, Sweden; 2 PO Hematology, Karolinska University Hospital, Stockholm, Sweden; 3 Division of Clinical Chemistry, Dept. of Laboratory Medicine, H5, Karolinska Institutet, Stockholm, Sweden; 4 Division of Hematology, Dept. of Medicine, Huddinge, Karolinska Institutet, Stockholm, Sweden; 5 Dept. of Cellular Therapy and Allogeneic Stem Cell Transplantation, Karolinska University Hospital, Stockholm, Sweden

**Keywords:** Autologous stem cell transplantation, ASCT, BEAM, Lymphoma, Real-world, Survival

## Abstract

**Background:**

Autologous stem-cell transplantation (ASCT) is a common treatment for lymphoma but it has some mortality.

**Methods:**

All 433 lymphoma patients who underwent ASCT for lymphoma at Karolinska Huddinge 1994–2016 were investigated, including CD34^+^ cell amounts, medications, infectious and other complications, intensive care, longitudinal laboratory values, and secondary myeloid neoplasia.

**Results:**

The 100-day non-relapse and overall mortalities were 5.6% and 7.2%. Stem-cell harvests < 5 million CD34^+^ cells/kg correlated with inferior 100-day and long-term survival. Prior to conditioning (93% BEAM), elevated (both 3–9 and ≥ 10 mg/L) C-reactive protein (CRP) and creatinine, and low albumin (but not higher age) predicted inferior higher 100-day survival. Intravenous antibiotics were given to 97% (22% positive blood cultures) and parenteral nutrition to 89%. After 1 year, 86% had normalized hemoglobin. The 5-year risk for secondary myeloid neoplasia was 4.1%, associated with smaller harvests.

**Conclusions:**

Before starting conditioning, patients should have preferably harvested ≥ 5 million CD34^+^ cells/kg and normal CRP, albumin, and creatinine. It appears safe to transplant patients ≥ 66 years.

**Electronic supplementary material:**

The online version of this article (10.1186/s40164-019-0131-3) contains supplementary material, which is available to authorized users.

## Background

Autologous stem-cell transplantation (ASCT) is part of standard therapy for lymphoma. Although ASCT appears to improve survival in several lymphomas [1–7], it is associated with a significant treatment-related mortality (TRM; 2.5% to 11%) [6, 8–10]. Selecting the right patient is essential.

Since ASCT was introduced more than 30 years ago, studies have suggested that outcome might be improved by conditioning with BEAM or BEAC instead of cyclophosphamide-total body irradiation [11] (although recent data are less persuasive [12]), peripheral instead of bone-marrow stem cells [13], and rituximab in pre-ASCT treatments for patients with CD20^+^ lymphoma [14]. High age has been proposed as a risk-factor for TRM [15]; however, newer data indicate that it is not an independent factor [10, 16, 17].

After decades of ASCT as standard practice and thousands of patients undergoing ASCT worldwide every year, no study has yet systematically explored the predictive role of established pre-transplant clinical and laboratory parameters to identify those at excessive risk for TRM or non-relapse mortality (NRM). Although therapy for several lymphoma subtypes has improved enormously in the last 15 years, ASCT remains an important modality [18, 19]. There is a need to identify robust predictors for TRM.

In our large, consecutive, population-based, unselected, detailed, real-world cohort, we have comprehensively studied clinical factors, laboratory parameters and supportive care to identify predictors for short- and long-term mortality (particularly NRM) in all 433 lymphoma patients who underwent ASCT at Karolinska University Hospital Huddinge between 1994 and 2016. In this report, we present factors that identify patients with excessive risks for NRM, and we show that histological subtypes of lymphoma are irrelevant for NRM.

## Methods

This paper describes all lymphoma patients who underwent ASCT at the Hematology Center, Karolinska Huddinge from January 1994 until December 2016. The material is population-based, because the Hematology Center was the only referral site for the procedure in South Stockholm County, and in January 2009 it became the only ASCT site in Stockholm Healthcare Region. Over time, there was a widening of indications and age-limit restrictions for the procedure: in the ‘90 s there was a sharp 65-year age-limit, in the ‘00 s a 70-year age-limit, and from 2010 a more individual approach based on biological age. Mandatory cardiac or pulmonary investigations were not conducted before the ASCT, but all patients were examined by an experienced hematologist before starting conditioning; a good performance status (ECOG ≤ 1) was a prerequisite, and no infections were allowed. Patients underwent dental sanitation before the procedure. Originally, the majority of patients were relapsed and transformed lymphomas. Upfront ASCT (in first remission) was at first only performed in transformed indolent lymphomas, but became more common over time, starting in 2000 with mantle cell lymphoma (the MCL-2 trial [4]), followed in 2003 with T cell lymphomas (the NLG-T-01 trial [5]) and in 2010 with primary CNS lymphoma (a regionwide change in practice). The stem-cell harvest targets were between 1994 and 1998 1–2, between 1999 and 2001 5 (but not another harvest day when 2 had been reached), during 2002 5 (but not another harvest day when 4 had been reached), and from 2003 5 million CD34^+^ cells/kg patient. These targets were based on local experience. Peripheral harvest-failures were addressed using bone-marrow harvests until 2005 and after that year plerixafor was routinely used according to a local algorithm. This study was approved by the Ethics Committee, Stockholm (Ref no. 2012/783-31/3 with Amendments 2015/327-32 and 2016/2379-32).

### Conditioning regimens and prophylaxis against infections and nausea

The first patient in 1994 who underwent ASCT received cyclophosphamide-total body irradiation as conditioning, and thereafter BEAM was the routine (93%). In a handful of cases BEAC was used (mostly because of occasional problems with melphalan supply). For CNS lymphoma BCNU-thiotepa was used from September 2015. Descriptions of these regimens are found in Supplement. They are all highly emetogenic and severely affect the patients’ nutritional intake, due to chemotherapy-induced nausea and vomiting as well as subsequent mucositis. In March 2015 aprepitant (for all 6 days of BEAM) was added to ondansetron and betamethasone as routine prophylaxis; a subset of patients given BEAM between January 2012 and August 2015 (n = 124) was investigated, to identify nausea and vomiting in records from nurses, doctors, dieticians and physiotherapists. Neither G-CSF nor other growth factors, nor antibiotic nor antifungal prophylaxis were routinely used. Prophylaxis were given to all patients against herpes viruses for at least three but up to 6 months and, starting at leukocyte engraftment, against *Pneumocystis jirovecii* for 6 months. Post-ASCT follow-up routines and vaccinations are presented in Additional file 1.

### Statistical methods

**Fig. 1:**
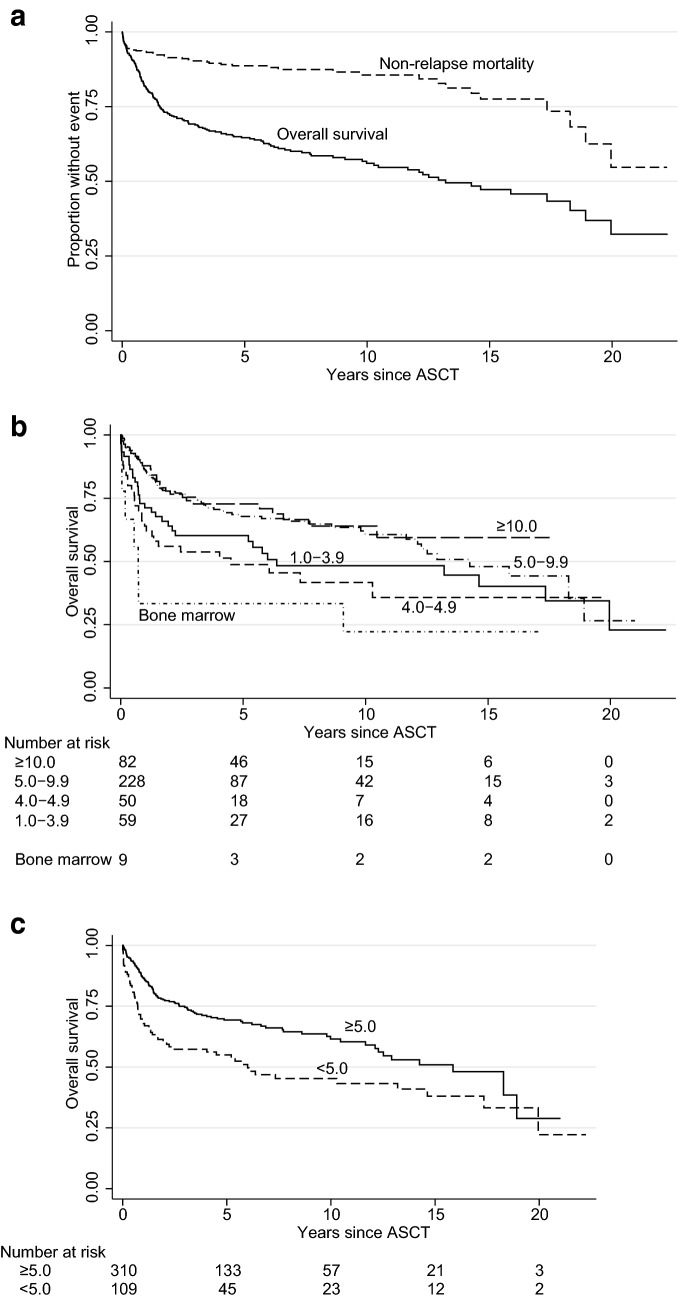
Long-term survival after autologous stem-cell transplantation (ASCT): **a** overall survival and non-relapse mortality, **b** overall survival by harvest yield in millions of CD34^+^ cells/kg patient (with bone-marrow harvested patients included as a separate category) and, **c** overall survival using the 5 million CD34^+^ cells/kg patient cut point

Patients were followed from ASCT until death or last follow-up (January 2018). In surviving patients, the median follow-up time was 6.0 years (range 1.2–22.2); three patients moved away from the Stockholm Region 4 months after their ASCT and were lost to follow-up. Depending on the nature of the variables, relationships between them were investigated using the χ^2^, Wilcoxon or Spearman test. OS was calculated from the day of ASCT until the day of death (Fig. 1a). Additional types of survival were also analyzed: NRM (counting only deaths that did not occur after lymphoma relapse, as defined by the EBMT), lymphoma and non-lymphoma mortality. Other time-to-event studies were performed, such as time to leukemia, normal platelets or parenteral nutrition. Univariate and multivariate analyses were conducted using Kaplan–Meier curves and Cox regression; the proportional hazards assumption was checked with graphs based on Schoenfeld residuals. Predictors for 100-day mortality were investigated using univariate and multivariate logistic regression. All *P* values are two-tailed and calculated using Stata 14.2 (StataCorp, College Station, TX, USA). *P *< 0.05 was considered significant. Cut-offs for laboratory values were based on the Karolinska Laboratory’s reference ranges. “Poor remission” before ASCT was arbitrarily decided by an experienced hematologist (B.E.W.) when assessing the last radiological and/or biopsy results prior to ASCT.

## Results

### Stem-cell harvests

424 patients had a peripheral stem-cell harvest sufficient for ASCT. The remaining nine were transplanted using bone-marrow harvested cells, eight of whom had failed prior peripheral harvests and one patient used bone-marrow harvested cells obtained 9 years before the ASCT. The outcomes were poor with bone-marrow harvested grafts (Fig. 1b), and they are not included in any analyses with peripheral harvest and given stem-cell amounts. While there was a clear prognostic threshold at 5 million CD34^+^ cells/kg (Table 1), there was no difference in 100-day non-relapse or overall mortality between 1.0 and 3.9 and 4.0–4.9, nor between 5.0 and 9.9 and ≥ 10.0 million peripheral CD34^+^ cells/kg. The 5 million cut-off was also significant in long-term overall survival (OS) analysis (Fig. 1c; *p *= 0.001), and also for long-term NRM (*p *= 0.013). In most patients (91%), the harvested and given dose was identical, but in 35 patients, the yields were so large that only half was used for transplantation. These 35 patients had harvested a median of 11.9 million (range 6.6–36.0) and had been rescued with a median of 6.0 million (range 3.4–25.3) cells CD34^+^ cells/kg; 10/35 were given < 5 million CD34^+^ cells/kg and they showed no inferior OS (*p *= 0.62) compared with those given ≥ 5 million; among these 35 patients there was only one death within 100 days after ASCT. Furthermore, in these 35 patients, the actual given dose did not correlate with time to leukocyte engraftment (*p *= 0.27) nor with normalisation of platelets (*p *= 0.98). Therefore, the harvested amount seemed more important than the given amount, as it appeared to also capture the prognostic properties of the underlying bone-marrow function. In subsequent analyses, we used a 5 million cutoff for harvest yield. Independent predictors for a harvest < 5 million peripheral CD34^+^ cells/kg were age > 45 years, harvest in the 1990s and relapsed/refractory disease.

**Table 1:** Clinical characteristics before ASCT

Characteristic	*N*	Per cent	OS 100 days		NRM 100 days	
	433	100%	OR (95% CI)	*p*	OR (95% CI)	*p*
Years of age, median (range; p10; p90): 56 (18–72; 35; 67)						
18–45	105	24		0.28		0.29
46–55	98	23				
56–65	165	38				
66–72	65	15				
Female sex	151	35		0.39		0.26
ASCT year						
1994–1999	42	10		0.43		0.25
2000–2009	161	37				
2010–2016	230	53				
Indication for ASCT						
Planned upfront	155	36		0.054		0.053
Relapsed/refractory	278	64				
Years since lymphoma diagnosis, median (range): 1.2 (0.2–22.6)				0.45		0.40
Number of prior lines of therapy						
1	161	40		0.16		0.20
2	201	50				
≥ 3	44	11				
Days between date of last chemotherapy and date of ASCT, median (range; p10; p90): 35 (20–415; 27; 53)						
< 28	54	14		0.79		0.48
28–34	135	34				
35–41	113	28				
42–55	63	16				
≥ 56	33	8				
Prior rituximab	263	61		0.069		0.12
Prior fludarabine	26	6		0.91		0.72
ASCT in poor remission	70	16		0.32		0.90
Harvested CD34^+^ cells/kg, median (range; p10; p90): 6.2 (1.0–48.0; 3.4; 13.7)						
1.0–3.9 million	59	14	2.0 (0.7–6.2)	0.040	3.4 (1.0–11.4)	0.040
4.0–4.9 million	50	12	4.2 (1.6–11.2)		5.2 (1.6–16.9)	
5.0–9.9 million	228	54	1		1	
10.0–48.0 million	82	20	1.4 (0.5–4.3)		1.9 (0.5–6.9)	
< 5 vs ≥ 5 million			3.1 (1.5–6.5)	0.003	4.0 (1.7–9.4)	0.001
Stem cell source						
Peripheral blood	424	98	1	0.008	1	0.002
Bone marrow	9	2	7.1 (1.7–29.8)		9.4 (2.2–40.3)	
Leukocytes per nL, median (range; p10, p90): 4.1 (1.0–25.9; 2.2; 7.2)						
Normal or high (≥ 3.5)	271	65		0.062		0.059
Neutrophils per nL, median (range; p10; p90): 2.4 (< 0.2–45.0; 1.0; 5.1)						
Normal or high (≥ 1.5)	246	76		0.13		0.26
Lymphocytes per nL, median (range; p10; p90): 0.8 (0.3–8.3; 0.4; 1.9)						
Normal or high (≥ 1.0)	65	37		0.65		0.40
Platelets per nL, median (range; p10; p90): 199 (7–1395; 105; 363)						
Low (< 150)	114	27	3.2 (1.5–6.6)	0.002	4.9 (2.1–11.7)	0.0003
Haemoglobin in g/L, median (range; p10; p90): 105 (75–150; 91; 123)						
Low (< 120)	364	87		0.25		0.45
Creatinine in multiples of UNL, median (range; p10; p90): 0.74 (0.29–1.90; 0.58; 1.02)						
Elevated (≥ UNL)	50	12	2.8 (1.2–6.8)	0.018	4.1 (1.6–10.1)	0.002
Albumin in g/L, median (range; p10; p90): 37 (14–44; 32; 41)						
Low (< 36)	102	26	2.6 (1.2–5.7)	0.016	3.2 (1.3–7.9)	0.013
C-reactive protein in mg/L, median (range; p10; p90): 2 (0–169; 0; 20)						
< 3	160	50	1	0.0006	1	0.0013
3–9	83	26	5.6 (1.4–21.6)		6.3 (1.2–31.9)	
≥ 10	75	24	9.0 (2.4–33.3)		11.0 (2.3–52.5)	
Conditioning						
BEAM	403	93		0.61		0.80
BEAC	22	5				
BCNU-thiotepa	7	2				
Cy-TBI	1	0				
Given dose of CD34^+^ cells/kg, median (range; p10; p90): 6.0 (1.0–48.0; 3.4; 11.4)						
1.0–3.9 million	61	15		0.087		0.071
4.0–4.9 million	58	14				
5.0–9.9 million	233	56				
10.0–48.0 million	66	16				
Tandem-treatment after ASCT	45	10		1.0		1.0
Local irradiation	22	5				
Splenectomy	2	0				
Temozolomide maintenance	18	4				
Allogeneic SCT	3	1				

OS, overall survival; NLM, non-lymphoma mortality; ASCT, autologous stem-cell transplantation; UNL, upper normal limit; HR, hazard ratio; CI, confidence interval; OR, odds ratio

### 100-day mortality

There were 24 cases of 100-day NRM (all treatment-related), and seven additional patients died from rapidly relapsing lymphoma, making overall 100-day mortality 31/433. The causes of NRM were sepsis (n = 12), non-bacterial infection (n = 2 [aspergilloma and zoster]), organ failure (n = 3 [one each from veno-occlusive disease, multiorgan and acute cardiac failure]) and combinations (n = 7). Multivariate models of 100-day non-relapse and overall mortality, are shown in Table 2: low harvest yields, low albumin, elevated CRP, and elevated creatinine were important. In patients with CRP < 3, 3–9, and ≥ 10 mg/mL 100-day NRM was 1.3%, 7.4%, and 12.3%, respectively. CRP ≥ 3 mg/L did not significantly correlate with remission status before ASCT, nor with type of lymphoma, nor with age, but was more common in patients with relapsed/refractory lymphoma (58% v 38%; p < 0.00005); however, relapsed/refractory disease was irrelevant when competing with CRP in multivariate analyses for 100-day non-relapse and overall mortality. Because only 318 patients had information on exact CRP (low CRP levels were reported as < 10 mg/L until 2007), we did a second multivariate analysis with 10 mg/L as a dichotomous threshold (and information in more patients; n = 402), and CRP was again independent (*p *= 0.044 for both 100-day NRM and OS).

**Table 2:** Multivariate models of factors before ASCT for non-relapse and overall mortality at 100 days

Factor	Non-relapse mortality		Overall mortality	
	OR (95% CI)	*p*	OR (95% CI)	*p*
Harvest < 5 million CD34 +/kg	3.6 (1.1–11.2)	0.028	2.5 (0.9–6.7)	0.063
Albumin < 36 g/L	2.9 (0.9–9.4)	0.079	3.3 (1.3–9.0)	0.017
C-reactive protein				
3–9 mg/L	9.7 (1.1–87.1)	0.042	6.1 (1.2–30.2)	0.027
≥10 mg/L	13.8 (1.6–119.0)	0.017	8.6 (1.8–41.9)	0.007
Elevated creatinine	4.3 (1.3–14.4)	0.019	2.6 (0.8–7.9)	0.098

The 100-day NRM was 17% in the 22% of patients who prior to conditioning had two or more factors of poor harvest, low albumin, elevated creatinine, and elevated CRP, 6% in the 37% with one factor and 0% in those 42% with none (*p *< 0.00005). The 100-day NRM decreased over calendar periods (Table 3), but the difference was not significant. Age had no bearing on 100-day NRM (Table 1), neither had the histological subtype of lymphoma (*p *= 0.64). Overall, non-relapse and lymphoma mortalities by calendar period and at 100 days, 6 months, and 1 year are shown in Table 3.

**Table 3:** Calendar periods and distributions of lymphoma diagnoses, selected characteristics, and mortality

Category	1994–1999		2000–2009		2010–2016		Total	
	*N*	%	*N*	%	*N*	%	*N*	%
Aggressive B-cell lymphoma	10	23.8	46	28.6	49	21.3	105	24.3
Indolent lymphoma	14	33.3	47	29.2	46	20.0	107	24.7
Of which transformed	4	28.6	31	66.0	35	76.1	70	65.4
Mantle cell lymphoma	2	4.8	27	16.8	55	23.9	84	19.4
T-cell lymphoma	6	14.3	23	14.3	37	16.1	66	15.2
Hodgkin lymphoma	10	23.8	18	11.2	23	10.0	51	11.8
Primary CNS lymphoma	0	0	0	0	20	8.7	20	4.6
Harvest < 5 million CD34 +/kg	22	64.7	36	23.2	51	22.2	109	26.0
Upfront transplantation	3	7.1	46	28.6	106	46.1	155	35.8
Age > 65 years	0	0	19	11.8	66	28.7	85	19.6
100-day overall mortality	4	9.5	14	8.7	13	5.7	31	7.2
100-day non-relapse mortality	4	9.5	11	7.0	9	4.0	24	5.6
100-day lymphoma mortality	0	0	3	2.0	4	1.8	7	1.7
6-month overall mortality^a^		14.3		13.1		7.4		10.2
6-month non-relapse mortality^a^		11.9		6.0		3.9		6.3
6-month lymphoma mortality^a^		2.7		5.3		3.6		4.2
1-year overall mortality^a^		28.6		19.9		16.6		19.0
1-year non-relapse mortality^a^		17.1		6.8		4.4		7.6
1-year lymphoma mortality^a^		13.8		11.5		12.8		12.4

^a^These values are estimated using survival analysis, why only percentages can be extracted

### Long-term NRM

At last follow-up 178/433 patients were dead (106 from lymphoma, 28 from ASCT complications, and 44 from other causes). Median OS after ASCT was 13.2 years (Fig. 1).

Progression/relapse of lymphoma after ASCT was seen in 173/433 patients. NRM had not reached the median at last follow-up (Fig. 1), while median survival from non-lymphoma death was 20.0 years. At 5 years the cumulative non-relapse and overall mortality was 14% and 35%. At 10 years, non-relapse and overall mortality was 20% and 44%. Associations between clinical factors and long-term OS and NRM are shown in Supplement.

**Fig. 2:**
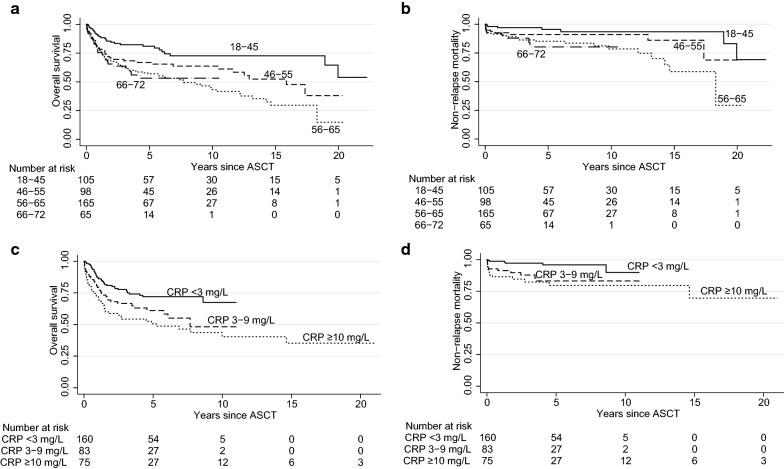
Overall survival (**a**) and non-relapse mortality (**b**) by age (in years) and overall survival (**c**) and non-relapse mortality (**d**) by CRP

The time between last chemotherapy and ASCT (median 35 days) did not correlate with short- or long-term NRM. C-reactive protein (CRP) levels were lower (*p *= 0.036) and albumin (*p *= 0.023) levels higher in patients with ≥ 35 days between last chemotherapy and ASCT. The histological subtypes of lymphoma did not correlate with long-term NRM (*p *= 0.83). Independent predictors for inferior long-term NRM were increasing age (*p *= 0.024), harvest < 5 million CD34^+^ cells/kg (*p *= 0.020), and increasing CRP (*p *= 0.004; Fig. 2; multivariate model in Additional file 1).

### Duration of leuko- and neutropenia

**Fig. 3:**
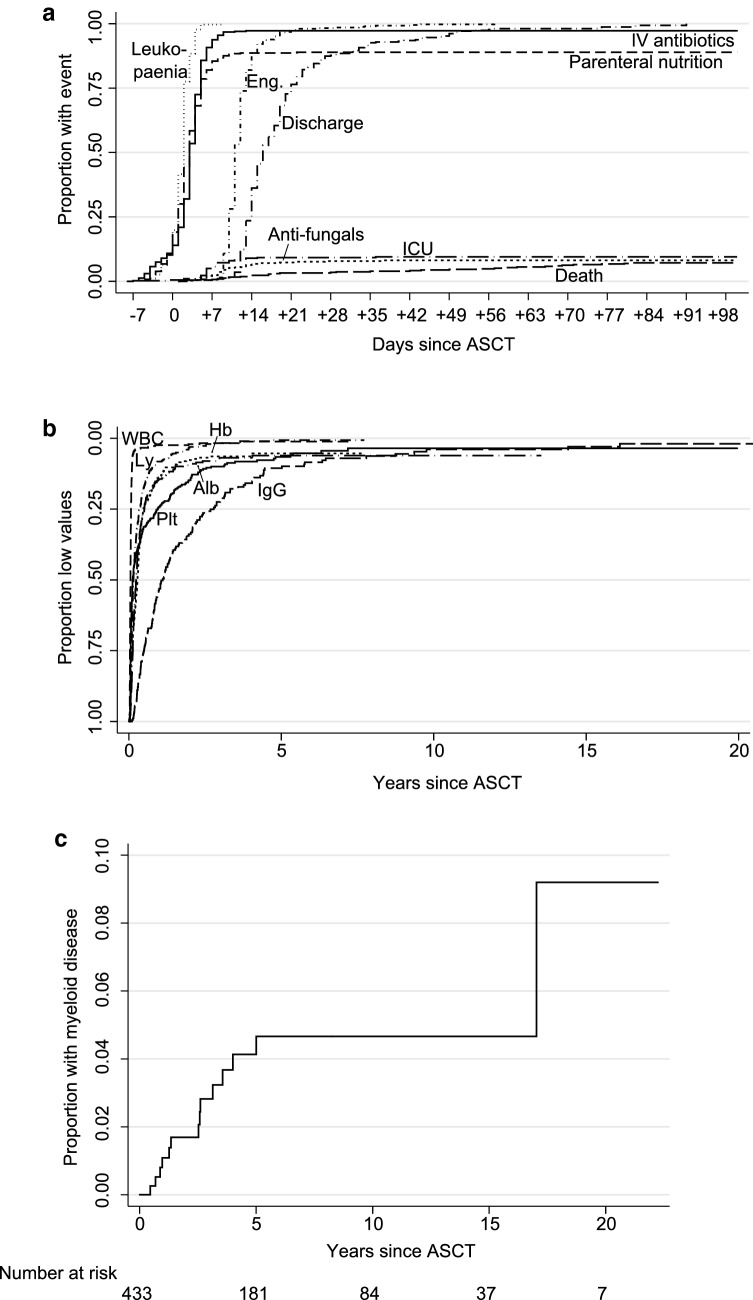
Time to **a** events within the first 100 days, **b** normalisation of laboratory values and **c** myeloid disease. Abbreviations: ASCT, autologous stem-cell transplantation; ICU, intensive care unit; WBC, leukocytes; Hb, hemoglobin; Ly, lymphocytes; Alb, albumin; Plt, platelets. Patients who died before the event are censored. “Leukopenia” and “Eng.” denote leukocytes passing below and above/equal 1.0/nL

Leukocytes and neutrophil changes showed identical patterns, and because the former had been more frequently measured, we focused on leukopenia (< 1.0/nL) and leukocyte engraftment (≥ 1.0/nL). Leukopenia occurred at median on day +2 after ASCT (p25, +1; p75, +2; range, -5 to +9) and median engraftment on day +11 (p25, +10; p75, +13), with a median duration of 10 days (p25, 8; p75, 12; range 0–25) of leukopenia (Fig. 3a). The median days for neutropenia (< 0.5/nL), neutrocyte engraftment (≥ 0.5/nL), and duration of neutropenia were identical. Good harvest yields and older age were independent predictors for faster time to leukocyte engraftment (Additional file 1: Table S1).

### Onset of fever, intravenous antibiotics, parenteral nutrition and intensive care

An infection occurring between start of conditioning and engraftment invariably prompted central and peripheral blood cultures and initiation of intravenous broad-spectrum antibiotics. Drug records could be obtained in 401/433 patients. 389 patients (97%) received intravenous antibiotics (median, day +3 [p25, +2; p75, +5]; Fig. 3a). In multivariate analysis, abnormal creatinine, albumin, and CRP were independent predictors for intravenous antibiotics (Additional file 1: Table S1). Significant bacteriemiae were verified in blood cultures from 88/402 patients (22%). Almost all bacteria were species normally found in the orogastrointestinal tract. There were no significant relations between 100-day mortality and positive blood cultures or bacterial species. A detailed description of the types of bacterial, fungal and viral infections is found in Supplement. Parenteral nutrition was given to 89% of the patients, the median start was day +3 (p25, +1; p75, +5). There was a strong linear correlation between the start dates of intravenous antibiotics and nutrition (*p *< 0.00005); 65% of the patients were given both therapies within 2 days of one another, and all patients who received parenteral nutrition also received intravenous antibiotics. With respect to time to parental nutrition, the only independent predictor was female sex; 94% of women and 86% of men received parenteral nutrition (*p *= 0.014). Therapeutic antifungals (excluding prophylaxis) were given to 31 patients (8.0%); median, day +9 (p25, +6; p75, +13). For antifungals, there was no independent predictor (but they co-varied with ICU admissions; *p *= 0.004). Patients who received antifungal therapy showed inferior 100-day non-relapse and overall mortality (*p *= 0.006 and *p *= 0.038), both at 16%. Thirty-eight patients (9.5%) were admitted to the intensive care unit (ICU) because of severe organ failure (median, day +6 [p25, +6; p75, +8]), and 16/38 ICU patients (42%) died within 100 days. The 100-day mortality was 28%, 33% and 73% in patients who were 0–2, 3–7, and 8–61 days in ICU, respectively (*p *= 0.028). High creatinine and low albumin correlated independently with risk for ICU admission.

### Nausea

Aprepitant reduced the proportion of patients experiencing acute nausea (from 49% to 20%; *p *= 0.002) and vomiting (from 45% to 5%; *p *< 0.0005) during BEAM and 24 h thereafter. There was a trend towards fewer days of parenteral nutrition with aprepitant (*p *= 0.066). There was no indication of additional toxicity with 6 days of aprepitant.

### Discharge and return to normality

The median patient was discharged on day +16 (range day +2 to + 99). Twenty-two patients died before discharge. Higher age, female sex, ASCT conducted in the 1990s, low platelets and low albumin independently predicted longer hospital stay (table in Supplement). The median man was discharged 2 days earlier than the median woman, and the median patient in the 2010s 10 days earlier than the median patient in the 1990s; the latter difference decreased to 4 days after the exclusion of bone-marrow harvests. For 421/433 (97%) longitudinal chemistry information was available. Leukocytes and neutrophils showed identical courses with a fast recovery to normal range (both median, day +19), while immunoglobulin G took longer (median, day +405). Normalisation of blood parameters are shown in Fig. 3b and with a table in Supplement. Time to normal platelet counts was significantly longer in patients with a peripheral stem-cell harvest < 5 million CD34^+^/kg (median 231 vs 36 days, *p *< 0.00005). Independent predictors for slow platelet normalisation were harvest yield, high creatinine and low platelets before the ASCT.

### Subsequent myeloid disease and allogeneic transplantation

Fourteen out of 433 patients developed subsequent myeloid disease: 7 acute myeloid leukemia (AML), 6 myeloid dysplastic syndrome (MDS) and 1 severe aplastic anemia. The median time from ASCT to myeloid disease was 2.6 years. The first case occurred 167 days after ASCT, and only a single case was seen later than 5 years (AML after 17 years); the cumulative 5-year risk was 4.1% (Fig. 3c). These 14 had significantly smaller stem-cell harvests than others (median 5.0 vs 6.3 million CD34^+^/kg; *p *= 0.028). The only independent predictor for developing myeloid disease was peripheral harvest yield as a continuous variable (table in Supplement). Only 3/14 (2 AML, 1 MDS) were alive, of whom two had undergone allogeneic stem-cell transplantation while one patient was diagnosed with AML only 12 days before last follow-up. Relapse of lymphoma was observed in 4/14 patients prior to the myeloid neoplasia. In total, 7 myeloid disease (4 AML, 2 MDS and 1 aplastic anemia) patients underwent allogeneic stem-cell transplantation. An additional 33 patients underwent allogeneic transplantation for lymphoma: 20/33 (61%) were alive at last follow-up; in these, the median follow-up time (after allogeneic transplantation) was 5.0 years.

### Changes over time

Table 3 shows, over the years, how the fraction of patients transplanted with a peripheral stem-cell harvest < 5 million CD34^+^/kg decreased, the patient age increased (median and maximal age 49.5 and 64, 56 and 69, 59 and 72 years in the three periods, respectively), the share of upfront ASCTs increased (all *p *< 0.002). Also, subnormal albumin levels increased (20%; 22%; 31%: *p *= 0.042), while more patients had CRP < 10 mg/L (44%, 78%, 86%; *p *= 0.0004) and longer times between the dates of last chemotherapy and the dates of ASCT (median 31.5, 33, 37 days; *p *< 0.00005). Reflecting changing practices, mantle cell lymphoma became common, while indolent B and Hodgkin less so (Table 3).

## Discussion

This large, population-based, detailed, real-world, single-center study of all lymphoma patients who underwent ASCT at Karolinska Huddinge 1994–2016 presents several important findings. Firstly, it appears vital that patients be completely recovered from their preceding regimens and thus biologically ready for the ASCT; low albumin, elevated creatinine and, above all, CRP predicted inferior 100-day NRM: in patients with CRP < 3 mg/L it was 1.3%, compared with 7.4% if CRP 3–9 mg/L and 12.3% if CRP ≥ 10 mg/L. Secondly, a harvest of ≥ 5 million CD34^+^ cells/kg appears highly relevant for short- and long-term mortality. Thirdly, (selected) patients aged 66–72 are not at higher risk than those aged 46–65, and there is no significant risk for inferior outcome in those who delay the ASCT; on the contrary, they show more often show normal CRP and albumin levels. Improved antiemetic prophylaxis made BEAM more tolerable, as also indicated in another study [20].

Median OS was 13.2 years but median NRM was not reached (median non-lymphoma mortality was 20.0 years); median 100-day non-relapse and overall mortality was 5.6% and 7.2%. Our study agrees with recent reports which show that ASCT is feasible in patients > 65 years, [10, 16, 17]. Comorbidities, not age, impact early TRM in ASCT [21]. However, for long-term NRM, we did find that age predicted survival, along with harvest yields and CRP. That the age at ASCT becomes increasingly predictive with long-term follow-up is not surprising (Fig. 2), given the natural course of aging. Among these 433 patients, there were 75 older than 70 years at last follow-up.

We also show that dynamic measurements of inflammation and poor health, such as CRP [22], albumin, and creatinine are important for TRM. It has long been known that stem-cell doses ≥ 5 million CD34^+^ cells/kg correlate with improved resource utilization [23]. We also show, as others before [24], that higher CD34^+^ yields correlate with quicker engraftment. Furthermore, normalisation of platelets and hemoglobin was strongly influenced by yield. What is more, the harvest predicted short- and long-term survival, and risk for myeloid disease. These outcomes are probably partly due to patients’ intrinsic bone-marrow function. We have previously shown that the average number of harvest days to obtain 5 million cells/kg was between 1.4 and 1.8 in all age groups, and that 19% did not reach the target, but 65% of these still proceeded to ASCT [25]. Today, with plerixafor, fewer patients do not reach the 5 million target, and, after a careful assessment of pros and cons, we do transplant the relatively few patients who have a yield of 2–5 million/kg.

We show the 100-day NRM/TRM to be 0% in patients with no risk factors according to our multivariate model, 6% in those who have one risk factor, and 17% in those who have two or more. We will therefore adjust the algorithm for our ASCT programme: patients with abnormalities in albumin, CRP, or creatinine will have their ASCT postponed and may undergo investigations and therapy for underlying causes. However, we believe that 0% 100–day NRM is not a realistic goal, because that would probably mean an exclusion of too many marginal patients, of which the majority would benefit from the procedure.

This is a population-based study with long follow-up times (median 6 years), and only three patients lost to follow-up. This allows us to calculate a 4.1% cumulative 5-year risk for developing subsequent myeloid disease. We show an association between that and smaller yields, and others have shown a correlation with MDS/AML and lower yields per harvest day [26], which suggests that there are underlying risk factors present before the ASCT. Indeed, preceding clonal hematopoiesis is often present before the ASCT (or prior to any therapy) in patients who develop secondary myeloid neoplasia [27–29].

We also made a careful description of infectious and other complications, useful for reference: 97% had intravenous antibiotics, 22% positive blood cultures, 89% parenteral nutrition; the median days for leukocyte engraftment and hospital discharge were +11 and +16. Invasive fungal infections and ICU admissions were rare (7.0% and 9.5%) but correlated with high mortality. Our focus has been on early, treatment-caused NRM, because overall survival (after the initial period) are mostly due to lymphoma relapses (and thus on histological subtypes, which are beyond the scope of this report), and after 5 years, strongly dependent on age (Fig. 2). Furthermore, our study was not designed to examine long-term toxicities, such as the previously reported moderate pulmonary and cardiac impairment [30, 31], or late solid tumors [32]. However, long-term survival from non-lymphoma mortality does not appear heavily impaired (median 20.0 years).

## Conclusions

We conclude that ASCT is feasible in (selected) patients 66–72 years and its treatment-related mortality might be minimized by using stem-cell harvests ≥ 5 million CD34^+^/kg, and by addressing abnormal CRP, creatinine, and albumin before starting conditioning.

## Additional file

**Additional file 1.** Supplemental information.

## Abbreviations

ASCT: autologous stem-cell transplantation
AML: acute myeloid leukemia
CRP: C-reactive protein
MDS: myeloid dysplastic syndrome
NRM: non-relapse mortality
OS: overall survival
TRM: treatment-related mortality
