# Supplementary material for: The Karolinska experience of autologous stem-cell transplantation for lymphoma: a population-based study of all 433 patients 1994–2016
Source: Exp Hematol Oncol. 2019 Mar 18;8:7. doi: 10.1186/s40164-019-0131-3 (PMC6423752; doi:10.1186/s40164-019-0131-3)
Supplement: Supplementary file 1 — Additional file 1. Supplemental information. [file 40164_2019_131_MOESM1_ESM.docx]

Descriptions of conditioning regimens

BEAM was dosed day -7: BCNU 300 mg/m2 4-hour infusion, intrathecal methotrexate 12 mg; days -6, -5, -4, -3: etoposide 200 mg/m2, cytarabine 400 mg/m2 12-hour infusion (day -3 also intrathecal methotrexate 12 mg); day -2: melphalan 140 mg/m2 (intrathecal methotrexate was omitted in patients with indolent, T-cell and Hodgkin lymphoma). BEAC was dosed -6: BCNU 300 mg/m2 4-hour infusion; days -5, -4, -3, -2: etoposide 100 mg/m2 twice daily, cytarabine 100 mg/m2 0.5-hour infusion twice daily, cyclophosphamide 35 mg/kg once and mesna 14 mg/kg four times daily. BCNU-thiotepa was dosed BCNU 400 mg/m2 1-hour infusion day -6; thiotepa 5 mg/kg twice daily day -5 and -4.

Post-ASCT vaccinations and follow-up routines

At three (or six if they had received rituximab) months patients started our vaccination program against pneumococci, diphtheria, tetanus and polio (and seasonal influenza). For clinical examinations, patients returned to the Hematology Center at one, three and six months, and then at least every sixth month for two years, then yearly for five years. After five years, patients continued their controls at their general practitioners’ who use the same region-wide electronic medical-records system as Karolinska (mantle cell and indolent lymphoma patients never ceased visiting the Hematology Center). Blood samples were obtained at each clinical control and in addition at least monthly for the first six months, and then at least every 3^rd^ month during the first two years. For this report, we accessed the patients’ medical records.

Significant associations between clinical factors and long-term OS and NRM

| Characteristic | |  | OS |  | NRM |  | |
| --- | --- | --- | --- | --- | --- | --- | --- |
|  |  |  | HR (95% CI) | *P* | HR (95% CI) | *P* | |
|  |  |  |  |  |  |  | |
| Age (years) | |  |  |  |  |  | |
|  | 18-45 |  | 1 | 0.0001 | 1 | 0.005 | |
|  | 46-55 |  | 1.9 (1.2-3.1) |  | 2.0 (0.8-5.3) |  | |
|  | 56-65 |  | 2.7 (1.8-4.3) |  | 4.1 (1.7-9.6) |  | |
|  | 66-72 |  | 2.7 (1.6-4.7) |  | 4.3 (1.5-12.2) |  | |
| ASCT year | |  |  |  |  |  | |
|  | 1994-1999 |  |  | 0.15 | 1 | 0.022 | |
|  | 2000-2009 |  |  |  | 0.6 (0.3-1.3) |  | |
|  | 2010-2016 |  |  |  | 0.3 (0.1-0.7) |  | |
| Indication for ASCT | | |  |  |  |  | |
|  | Planned upfront | | 1 | 0.0001 | 1 | 0.005 | |
|  | Relapsed/refractory | | 2.1 (1.5-2.9) |  | 2.8 (1.4-5.7) |  | |
| No. of prior lines of therapy | | |  |  |  |  | |
|  | 1 |  | 1 | 0.003 | 1 | 0.032 | |
|  | 2 |  | 1.8 (1.2-2.5) |  | 2.6 (1.2-5.4) |  | |
|  | ≥ 3 |  | 2.0 (1.2-3.4) |  | 3.0 (1.1-8.0) |  | |
| ASCT in poor remission | | | 2.2 (1.5-3.1) | < 0.00005 | | 0.83 | |
| Harvested CD34^+^ cells/kg | | |  |  | | |  |
|  | < 5 vs ≥ 5 million | | 1.7 (1.2-2.3) | 0.001 | 2.0 (1.2-3.6) | 0.013 | |
| Stem cell source | |  |  |  |  |  | |
|  | Peripheral blood | | 1 | 0.010 | 1 | 0.045 | |
|  | Bone marrow | | 2.7 (1.3-5.8) |  | 3.1 (1.0-10.7) |  | |
| Normal or high leukocytes | | | 1.6 (1.1-2.2) | 0.009 | 2.0 (1.0-3.9) | 0.045 | |
| Low platelets | | | 1.9 (1.4-2.6) | 0.0001 | 2.5 (1.5-4.4) | 0.001 | |
| Low hemoglobin | | | 1.8 (1.1-3.1) | 0.029 |  | 0.30 | |
| Elevated creatinine | | |  | 0.24 | 2.1 (1.1-4.3) | 0.034 | |
| Low albumin | | | 1.8 (1.3-2.5) | 0.001 | 2.5 (1.4-4.5) | 0.002 | |
| C-reactive protein (mg/L) | | |  |  | |  | |
|  | < 3 | | 1 | 0.0009 | 1 | 0.005 | |
|  | 3-9 |  | 1.7 (1.1-2.7) |  | 3.8 (1.4-10.2) |  | |
|  | ≥ 10 |  | 2.3 (1.5-3.5) |  | 4.9 (1.9-12.9) |  | |

Multivariate model of long-term non-relapse mortality

Factor Hazard ratio (95% confidence interval) *P*

Age (years) 0.024

18-45 1

46-55 3.9 (0.4-35.5)

56-65 10.7 (1.4-81.0)

66-72 13.5 (1.7-107.9

Harvest <5 million CD34+/kg 2.4 (1.4-5.0) 0.020

C-reactive protein (mg/L) 0.004

<3 1

3-9 3.6 (1.3-9.8)

≥10 5.1 (1.9-13.4)

| **Factors independent in multivariate analysis for different outcomes.** | | | | | |
| --- | --- | --- | --- | --- | --- |
|  |  |  |  |  |  |
| Outcome | | Independent factors | | HR | *P* |
|  |  |  |  |  |  |
|  | Time to intravenous antibiotics | |  |  |  |
|  |  | Elevated creatinine | | 1.7 | 0.004 |
|  |  | Low albumin | | 1.5 | 0.001 |
|  |  | C-reactive protein ≥3 g/L | | 1.3 | 0.020 |
|  | Time to parenteral nutrition | |  |  |  |
|  |  | Female sex |  | 1.5 | 0.0002 |
|  | Time to admission to intensive care unit | | |  |  |
|  |  | Elevated creatinine | | 2.2 | 0.038 |
|  |  | Low albumin | | 2.9 | 0.002 |
|  | Time to leukocyte engraftment* |  |  |  |  |
|  |  | Harvest < 5 million CD34+/kg | | 0.7 | 0.005 |
|  |  | Age > 55 years |  | 1.3 | 0.006 |
|  | Time to discharge from hospital* |  |  |  |  |
|  |  | ASCT in the 1990s | | 0.5 | 0.035 |
|  |  | Age > 55 years |  | 0.7 | 0.003 |
|  |  | Female sex |  | 0.7 | 0.008 |
|  |  | Low platelets | | 0.7 | 0.017 |
|  |  | Low albumin | | 0.6 | 0.00002 |
|  | Time to normal platelets* | |  |  |  |
|  |  | Harvest < 5 million CD34+/kg | | 0.6 | 0.001 |
|  |  | Low platelets | | 0.4 | < 0.00005 |
|  |  | Elevated creatinine | | 0.6 | 0.005 |
|  | Time to normal haemoglobin* | |  |  |  |
|  |  | Age > 55 |  | 0.6 | < 0.00005 |
|  |  | ASCT in the 1990s | | 0.5 | 0.002 |
|  |  | Relapsed/refractory disease | | 0.7 | 0.004 |
|  |  | Low platelets | | 0.7 | 0.007 |
|  |  | Haemoglobin < 120 g/L | | 0.6 | 0.003 |
|  | Time to myeloid neoplasia | |  |  |  |
|  |  | Harvest, per million CD34+/kg | | 0.8 | 0.034 |
|  |  |  |  |  |  |
| * Note that the event here is a good outcome, why adverse risk factors have hazard ratios < 1 (and favourable ones > 1).  Abbreviations: HR, hazard ratio; CI, confidence interval; ASCT, autologous stem-cell transplantation. | | | | | |

Types of infection

| **Significant* positive bacterial blood cultures.** | | | |  |
| --- | --- | --- | --- | --- |
|  |  |  |  |  |
|  | All cultures |  |  | N |
|  |  |  |  | 88 |
|  | Gram-positive | |  | 42 |
|  |  | Alpha Streptococci | | 21 |
|  |  | Beta Streptococci | | 1 |
|  |  | Enterococci | | 15 |
|  |  | Staphylococci | | 2 |
|  |  | Different G+ species** | | 3 |
|  | Gram-negative | |  | 39 |
|  |  | Escherichia coli |  | 16 |
|  |  | Enterobacter | | 11 |
|  |  | Klebsiella |  | 4 |
|  |  | Pseudomonas | | 4 |
|  |  | Capnocytophaga | | 1 |
|  |  | Moraxella |  | 1 |
|  |  | Different G- species*** | | 2 |
|  | Combinations of Gram-positive and -negative**** | | | 7 |
| * Insignificant blood cultures are findings deemed to be contaminations, mostly low-virulent S. epidermidis in only one dish. | | | | |
| ** All three combinations of Gram-positive species contained Enterococci, two with Alpha Streptococci and one with C. tertium. | | | | |
| *** One combination of Enterobacter and E. coli, one of Klebsiella and Capnocytophaga. | | | | |
| **** Five combinations of Alpha Streptococci and E. coli (one also with Enterobacter), one of Alpha Streptococci and Klebsiella, one of Enterococci and Pseudomonas. | | | | |

Significant bacteriemiae were verified in blood cultures from 88/402 patients (22%). Almost all bacteria were species normally found in the orogastrointestinal tract, as shown in the table below. There were no significant relations between 100-day mortality and positive blood cultures or bacterial species. Invasive fungal infection was diagnosed in 28 (7.0%) patients (13 clinically-radiologically [decided by the attending senior hematologist] and 15 in blood cultures), who showed a 21% 100-day NLM (P=0.0005). Clinical diagnoses of invasive fungal infection were as predictive for poor outcome as those made in blood cultures (100-day NLM 23% and 20%, respectively). The diagnosed cultures were 12 *Candida*, 1 *Aspergillus*, 1 *Fusarium*, 1 untyped fungus. Within the first 6 months after ASCT, 3 patients (0.8%) were diagnosed with *Pneumocystis jirovecii* pneumonia and 22 (5.1%) with herpes zoster.

| **Normalisation of laboratory parameters.** | | | | |  |  |  |  |
| --- | --- | --- | --- | --- | --- | --- | --- | --- |
|  | Per cent patients attaining normal values | | | | | | | |
|  | Leukocytes | Neutrophils | Lymphocytes | Platelets | Haemoglobin | Albumin | Immunoglobulin G | |
|  |  |  |  |  |  |  |  |  |
| Median time to normal value (days) | 19 | 19 | 54 | 46 | 103 | 85 | 405 |  |
| 100 days | 96.1% | 95.8% | 69.3% | 60.3% | 49.0% | 56.8% | 11.1% |  |
| 6 months | 96.7% | 98.4% | 83.3% | 68.8% | 75.4% | 74.2% | 25.6% |  |
| 1 year | 97.6% | 99.7% | 91.8% | 75.5% | 86.2% | 85.6% | 46.9% |  |
| 2 years | 97.9% | 100.0% | 97.0% | 85.2% | 92.3% | 90.5% | 66.1% |  |
| 5 years | 98.8% | 100.0% | 99.3% | 93.4% | 94.7% | 93.9% | 89.4% |  |
| 10 years | 100.0% | 100.0% | 100.0% | 96.5% | 100.0% | 93.9% | 96.1% |  |
| 20 years | 100.0% | 100.0% | 100.0% | 100.0% | 100.0% | 100.0% | 98.0% |  |
|  |  |  |  |  |  |  |  |  |
| Note: Normal values were defined as being as least as high as the following: leukocytes – 3.5/nL; neutrophils – 1.5/nL; lymphocytes – 1.0/nL; platelets – 150/nL; haemoglobin – 120 g/L; albumin – 35 g/L; immunoglobulin G – 6.7 g/L. | | | | | | | | |
|  |  |  |  |  |  |  |  |  |
